# Galectin-3: a key player in microglia-mediated neuroinflammation and Alzheimer's disease

**DOI:** 10.1186/s13578-021-00592-7

**Published:** 2021-04-27

**Authors:** Yinyin Tan, Yanqun Zheng, Daiwen Xu, Zhanfang Sun, Huan Yang, Qingqing Yin

**Affiliations:** 1 Department of Neurology, Shandong Provincial Hospital Affiliated to Shandong First Medical University, Jinan, 250021 Shandong China; 2 Department of Neurology, The Dongshan Hospital of Linyi, Linyi, 276017 Shandong China; 3 Department of Neurology, The People Hospital of Huaiyin Jinan, Jinan, 250021 Shandong China; 4 Department of Radiology, Shandong Provincial Hospital Affiliated to Shandong First Medical University, Jinan, 250021 Shandong China; 5 Department of Geriatric Neurology, Shandong Provincial Hospital Affiliated to Shandong First Medical University, Jinan, 250021 Shandong China

**Keywords:** Galectin-3, Microglia, Neuroinflammation, Amyloid-β, Alzheimer’s disease

## Abstract

Alzheimer’s disease (AD) is the most common cause of dementia and is characterized by the deposition of extracellular aggregates of amyloid-β (Aβ), the formation of intraneuronal tau neurofibrillary tangles and microglial activation-mediated neuroinflammation. One of the key molecules involved in microglial activation is galectin-3 (Gal-3). In recent years, extensive studies have dissected the mechanisms by which Gal-3 modulates microglial activation, impacting Aβ deposition, in both animal models and human studies. In this review article, we focus on the emerging role of Gal-3 in biology and pathobiology, including its origin, its functions in regulating microglial activation and neuroinflammation, and its emergence as a biomarker in AD and other neurodegenerative diseases. These aspects are important to elucidate the involvement of Gal-3 in AD pathogenesis and may provide novel insights into the use of Gal-3 for AD diagnosis and therapy.

## Background

Alzheimer’s disease (AD) is the most common form of dementia and is a neurodegenerative disease characterized by impaired cognition and behaviour in elderly people over 65 years of agen [1]. The deposition of extracellular neurotoxic plaques primarily composed of amyloid-β protein (Aβ) and intracellular hyperphosphorylated tau neurofibrillary tangles (NFTs) are both key histopathological hallmarks of AD [2]. In addition to Aβ deposition and tau aggregation, neuroinflammation has emerged as the third core feature and can act as a double-edged sword in the complex pathogenesis of AD [3]. Microglia are resident immune cells and play a central role in neuroinflammation [4]. Emerging evidence suggests that galectins, which are a 15-member family of evolutionarily conserved glycan-binding proteins, can act as endogenous modulators of neuroinflammation and potentially neurodegeneration [5, 6]. Among them, galectin-3 (Gal-3) is involved in cell adhesion, proliferation, migration, apoptosis, tumour progression, inflammation and innate and adaptative immune system modulation [7–9].

In the central nervous system (CNS), Gal-3 is deemed crucial for resident microglial activation and has been shown to play a key role in the pathology of AD [10]. Gal-3 expression was highly upregulated in the brains of AD patients and 5xFAD (familial AD) mice and found to be specifically expressed in microglia associated with Aβ plaques [11, 12]. In addition, Gal-3 is a ligand of endogenous triggering receptor expressed on myeloid cells 2 (TREM2), a key receptor driving microglial activation in AD [12]. Thus, Gal-3 is a central upstream regulator of the microglial immune response in AD. Here, we review the research progress in the knowledge of Gal-3, including its origin, its biological and pathobiological functions in AD, and the subsequent issues in clinical practice, such as use in AD diagnosis and therapy.

## Main text

### Gal-3: biochemistry and physiological function

#### The structure of Gal-3

**Fig. 1:**
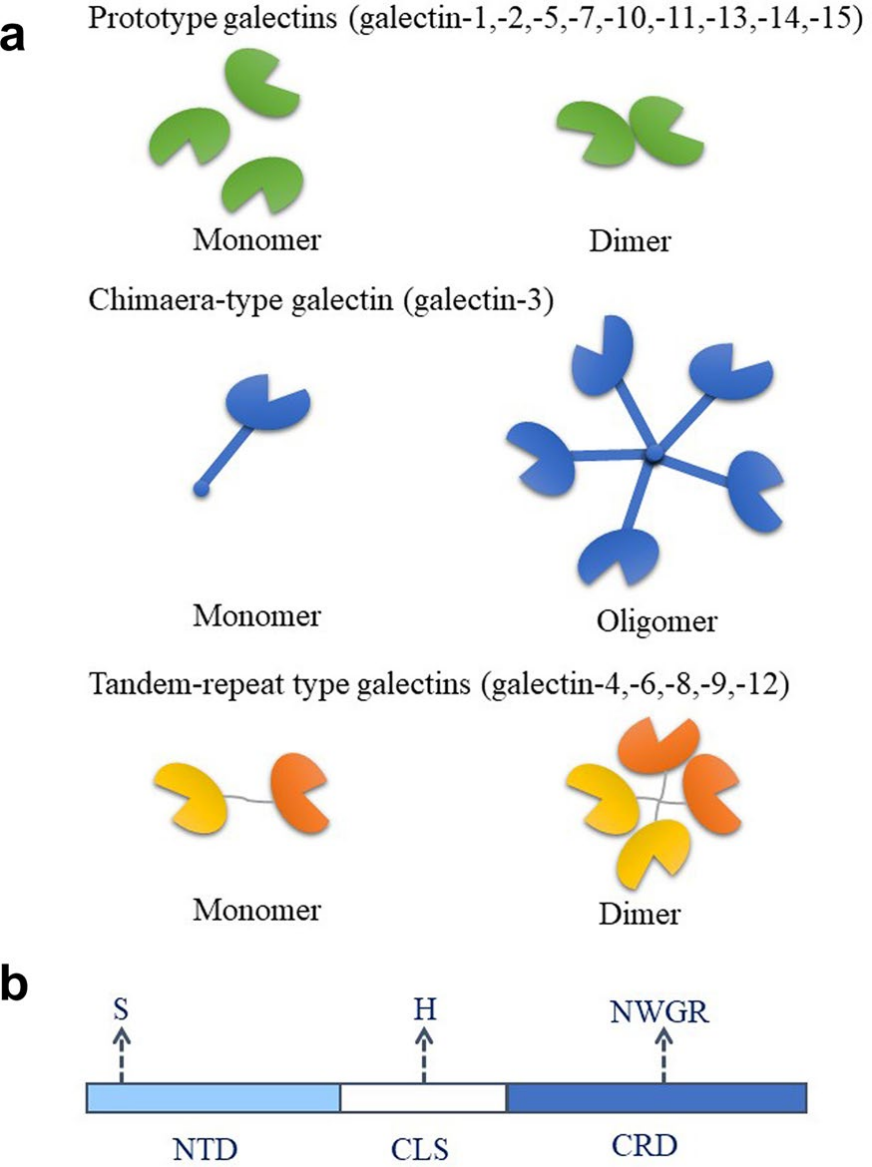
Galectin family classification and structure of galectin-3 (Gal-3). **a** Based on their structures, the galectin family has been subdivided into three subtypes: prototype galectins, chimaera-type galectins and tandem-repeat-type galectins. Some galectins can form dimers or oligomers. **b** The Gal-3 protein structure consists of an N-terminal domain (NTD), collagen-like sequence (CLS) and carbohydrate recognition domain (CRD)

Galectins consist of evolutionarily highly conserved carbohydrate-recognition domains (CRDs) containing approximately 130 amino acid sequences that bind β-galactose in glycoconjugates [13]. In mammalian tissues, a total of 15 galectins have been identified to date, and they have been subdivided into three subtypes according to their structures [5, 14]: the prototype galectins include the largest number of subgroups and contain a single CRD; the chimaera-type galectins contain a single CRD and an intrinsically disordered sequence at the N-terminal domain that promotes oligomerization; and the tandem-repeat type galectins consist of two CRD motifs (Fig. 1a).

Gal-3 is a unique chimaera-type member of the galectin family. The Gal-3 gene, named LGALS3 (human locus 14q21-22), is composed of six exons and five introns spanning approximately 17 kilobases [14–16]. There is a particular Gal-3 internal gene (GALIG) located in the second intron of LGALS3 [17]. Interestingly, Gal-3 and GALIG use the same coding sequences but alternative open reading frames during the process of transcription [18]. GALIG encodes two proteins, Cytogaligin and Mitogaligin, to contribute to apoptosis [19]. Gal-3 expression can be regulated by promoter hypermethylation of the LGALS3 gene and several elements in this promoter, including putative Sp1 binding sites (GC boxes), cAMP-dependent response element (CRE) motifs, sis-inducible element (SIE), nuclear factor-κB (NF-κB)-like sites and consensus basic helix–loop–helix (bHLH) core sequences [20]. Previous studies have shown that Gal-3 expression could be downregulated by miR-124-3p from osteocyte-derived exosomes [21] and Krüppel-like factor 3 (KLF-3) [22] and upregulated by Runt-related transcription factor 2 (Runx2) [23].

Gal-3 (m.w. 29–35 kDa) consists of a highly conserved CRD, a collagen-like sequence (CLS) and a relatively flexible N-terminal domain (NTD) [20, 24] (Fig. 1b). The NTD contains approximately 120 amino acids and consists of a tandem repeat of 7–14 amino acid residues rich in proline, tyrosine and glycine, which is essential for the full biological activity of Gal-3. There is an N-terminal region (NTR) of 12 amino acids in this NTD. It allows nuclear translocation and the decrease in affinity to its ligands via serine 6 phosphorylation by casein kinases 1 and 2 [25]. There is a CLS between the CRD and the NTD, containing approximately 100 amino acids. This sequence includes collagenase-cleavable H-domains and matrix metalloproteinases (MMPs) that act on histidine 64. The CRD, consisting of approximately 135 amino acid residues, is responsible for the entire carbohydrate-binding site and its interaction with glycoconjugates containing N‑acetyllactosamine. It retains lectin activity and can be proteolytically digested by trypsin [15]. Moreover, there is an Asp-Trp-Gly-Arg (NWGR) motif regulating antiapoptotic activity. This sequence could participate in the aggregation of Gal-3 molecules without ligands. Therefore, Gal-3 can bind proteins in carbohydrate‑dependent and carbohydrate‑independent ways.

### The secretion of Gal-3

Gal-3 was formerly named Mac-2 antigen [26], L-29 [27], IgE-binding protein [28], and CBP30 [29]. In 1982, Gal-3 was first identified in murine peritoneal macrophages and named Mac-2 antigen. Originally described in 1984 [30], Gal-3 was cloned and classified as a galactosidase-binding lectin in 1991 [31]. Gal-3 is synthesized on free ribosomes of the cytoplasm, and because it has no signal sequence, it cannot be translocated into the endoplasmic reticulum (ER) [32]. Although this molecule does not traverse the ER/Golgi network, emerging evidence has shown that Gal-3 also has an extracellular localization. Gal-3 does not utilize the classical secretory pathway through the ER/Golgi secretion system due to the lack of a signal sequence that controls its translocation and secretion in the classical pathway. This molecule has been shown to be secreted and exported from cells by a novel, alternative secretory pathway including specific vesicles and/or exosomes [32–34]. Modulation of Gal-3 secretion by exosomes is an interesting concept and potential therapeutic approach because our recent review shows that naturally occurring or engineered exosomes derived from stem cells, or potentially other cell types, provide therapeutic effects in AD [35, 36]. Moreover, recent experiments have shown that Gal-3 can shuttle between the cytoplasm and nucleus across the nuclear pore complex in a dynamic process [37]. The carboxyl terminal region (the last 28 amino acids) of Gal-3 plays an irreplaceable role in targeting the nucleus [38].

### The expression of Gal-3

Gal-3 is ubiquitously expressed in multiple human tissues and cells and is developmentally regulated. It is present in the heart, liver, kidney, chondrocytes and epithelia, including the skin, epithelium of the respiratory tract, digestive tract, and urinary tract in early embryogenesis [39]. In an adult organism, Gal-3 is widely expressed in epithelial cells, myeloid cells, and amoeboid cells. Gal-3 is also expressed in immune cells, such as macrophages, natural killer cells, T and B cells, neutrophils, and eosinophils, to participate in the immune response [40]. Furthermore, Gal-3 is mainly distributed in the nucleus, cytoplasm, extracellular space, and circulation [41]. As a multifunctional protein, Gal-3 plays a crucial role in numerous physiological and pathological processes, such as cell adhesion, activation, development, differentiation, apoptosis, immune responses, inflammation, angiogenesis, fibrogenesis and cancer progression [15, 20, 42]. Generally speaking, the biological functions of Gal-3 are dependent on its subcellular localization, different types of tissues and pathological state.

### The biological functions of Gal-3

Gal-3 predominantly exists in the cytoplasm and participates in cell proliferation, differentiation, apoptosis and survival through various signalling pathways. Studies have shown that Gal-3 activates the Wnt/β-catenin signalling pathway and then regulates cell proliferation, invasion, and migration [43, 44]. The interaction of Gal-3 and B-cell lymphoma-2 (Bcl-2) generates cell apoptotic events and restrains autophagy via the (TRPC1/4) signalling pathway and Akt phosphorylation of glycogen synthase kinase-3β (GSK-3β) [45, 46]. Gal-3 also modulates cell apoptosis by interacting with synexin [47], nucling [48] and CD95 (APO-1/Fas) [49]. Gal-3, expressed in the mitochondria, could obstruct cytochrome c release, contributing to inhibition of mitochondrial depolarization and oxidative damage [26]. The stability of the mitochondria prevents caspase activation and then suppresses cell apoptosis [50]. Gal-3 also plays a key role in maintaining mitochondrial function, such as restraining excessive mitochondrial fission, maintaining morphology and modulating the activity of respiratory chain complexes [51]. At the lysosomal level, Gal-3 coordinates lysosomal repair, removal and replacement by interacting with ALIX and the ESCRT-III effector CHMP4. During this repair process, Gal-3 induces autophagy and activates lysosomal biogenesis [52]. Gal-3 is also involved in abundant inflammatory reactions [42].

Several studies have confirmed that Gal-3 can shuttle between the cytoplasm and nucleus across the nuclear pore complex in a dynamic process [37]. Thus, Gal-3 plays essential roles in the nucleus. Previous immunofluorescence experiments have suggested that Gal-3 is diffusely distributed or is positioned on numerous discrete punctate structures called speckles in the nucleoplasm [53]. The interaction between Gal-3 and the homology domain of thyroid-specific transcription factor 1 (TTF-1) upregulates the transcriptional activity of TTF-1, finally facilitating the proliferation of thyroid cells [54]. Gal-3 colocalizes with two other proteins in speckles in the nucleoplasm, forming splicing complexes and participating in pre-mRNA splicing [55]. Gal-3 directly binds Gemin4, a component of the SMN (survival of motor neuron) protein complex, which facilitates pre-mRNA splicing machinery [56]. In addition, Gal-3 induces promoter activity of cyclin D1, a vital inducer of the cell cycle and a possible oncogene, through multiple cis-elements, revealing the growth-promoting effect of Gal-3 [57].

The extracellular biological activities of Gal-3 are mainly dependent on binding cell surface and extracellular matrix glycan-related ligands, such as laminin, MAC-2, collagen IV, fibronectin, Mac-2BP, and macrophage surface antigens [58]. These processes promote cell-to-cell and cell-to-matrix interactions, inflammatory responses, and cell development. The N-terminal domain of Gal-3 binds ligand (Bcl-2) and inhibits apoptosis [59]. Increased expression of Gal-3 in the extracellular space has already been verified in human diseases, including tumour development and progression and prognosis, neural degeneration and cardiovascular disease [60].

### Gal-3 and neuroinflammation

#### The expression of Gal-3 in normal brain

Gal-3 expression can modulate inflammatory response of the nervous system and is implicated in the pathogenesis of diverse neurological diseases. However, the proinflammatory or anti-inflammatory functions are dependent on different brain areas, injury conditions and stages of disease [9]. Gal-3 constitutive expression was found in neuronal tissues, neurons and glial cells in different brain regions [61]. Expression of Gal-3 has been observed in many brain regions of the normal adult rat by using immunohistochemistry: telencephalon (some parts of the cerebral cortex, the olfactory region, amygdaloid nucleus, the stria terminalis, the vascular organ of the lamina terminalis), diencephalon (thalamus, hypothalamus), brain stem and cerebellum (the inferior colliculus, the lateral parabrachial nucleus, the pontine nucleus, and cochlear nucleus and the fibres composing cerebellar peduncles). In the central nervous system, Gal-3 can be observed in microglia, astrocytes, and oligodendrocytes under neuroinflammatory conditions. Gal-3 is predominantly expressed by microglia and astrocytes in vitro [47].

### Gal-3 and microglia in neuroinflammation

Inflammation of the central nervous system can be divided into acute and chronic inflammation. Acute inflammation is an instant response to the appearance of damaging stimuli and is characterized by a prompt and efficient process to remove possibly destructive agents and rapidly resolve inflammation and maintain homeostasis. Chronic inflammation will subsequently occur when the host cannot reverse acute inflammation and is characterized by dysfunction or overreaction of microglia and disruption or even death of neurons and glial cells [62]. In turn, overactivated microglia accelerate the deterioration of nervous system damage. Activated microglia show active proliferating potential, morphological changes, migration to damage sites and cytokine production [63]. Activated microglia are the primary effectors in the inflammatory reaction of the central nervous system [64]. Due to the influence of the diverse microenvironment, microglia have dual phenotypes (proinflammatory M1 phenotype and anti-inflammatory M2 phenotype) and functional plasticity, which can dynamically transform into each other to ensure tissue homeostasis [65].

**Fig. 2:**
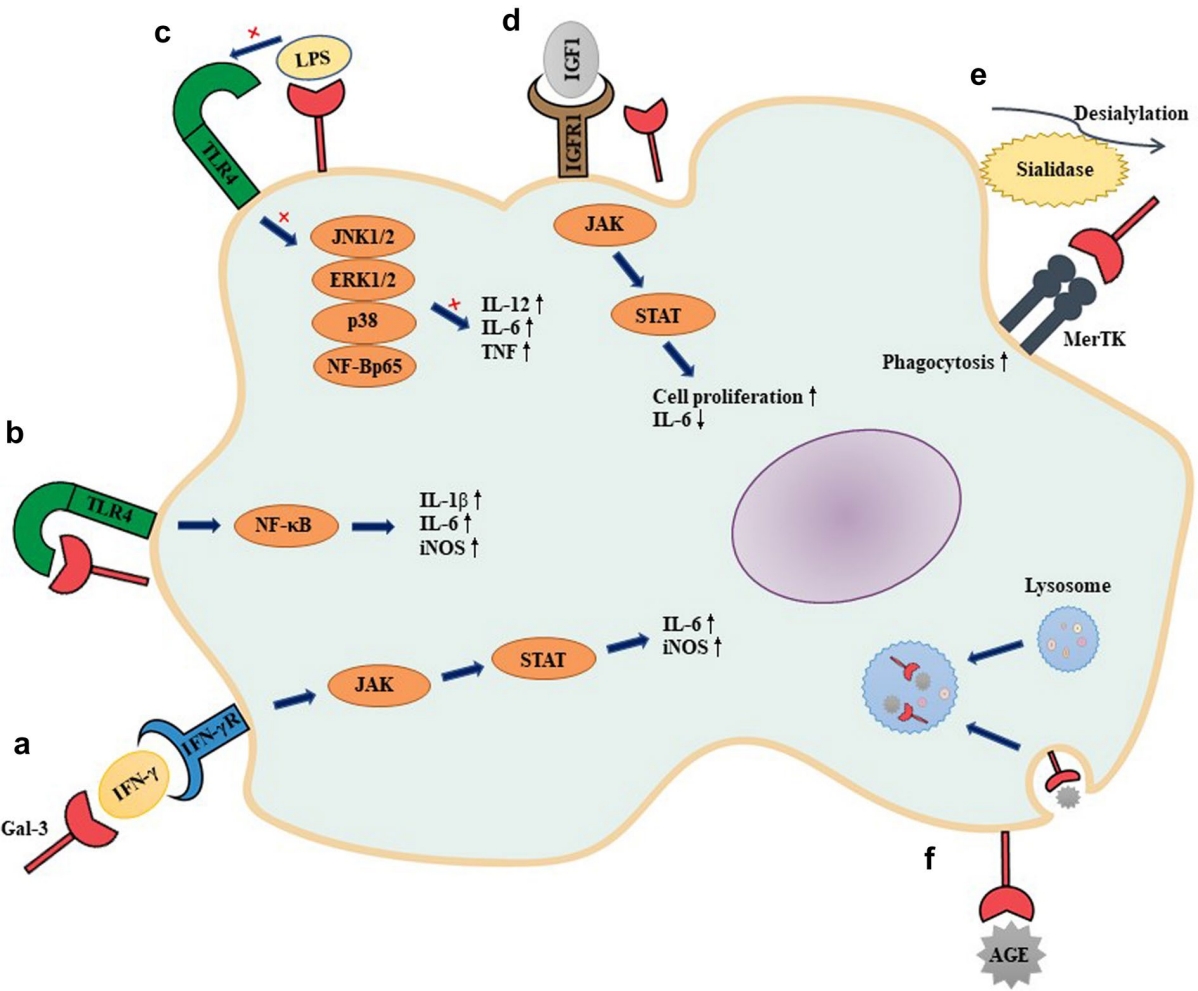
The molecular mechanism of microglial activation by galectin-3 (Gal-3). **a** Gal-3 activates microglia via IFN-γ and further induces the production of proinflammatory cytokines via the Janus kinase (JAK)/signal transducer and activator of transcription (STAT) pathway. **b** Gal-3 binds to microglial Toll-like receptor 4 (TLR4), triggering a proinflammatory response under acute neuroinflammatory conditions. **c** Gal-3 prevents lipopolysaccharide (LPS) from interacting with TLR4 by preferentially binding to LPS, which inhibits downstream proinflammatory cytokine production. **d** Gal-3/insulin-like growth factor 1 receptor (IGFR-1) interaction activates the IGF-mediated JAK/STAT pathway and microglial proliferation. **e** After secretion of sialidase to eliminate sialic acid from cell surface glycoproteins, Gal-3 binds the phagocytic receptor Mer tyrosine kinase (MerTK) and then contributes to phagocytosis by microglia. **f** Gal-3 interacts with advanced glycosylation end products (AGEs) to degrade its toxicity by fusing lysosomes

Gal-3 plays a crucial role in the neuroinflammatory response by mediating activated microglia (Fig. 2). In different brain-damaging contexts, Gal-3 regulates microglial M1/M2 polarization to ensure tissue homeostasis, and this process is accomplished via the specific interaction of lectin and glycan [6]. However, activation of microglia is regarded as a double-edged sword, and the changes in either proinflammatory or anti-inflammatory effects mediated by Gal-3 depend on the disease types, stages and severity [42, 66, 67].

Under inflamed conditions, the expression and release of Gal-3 can be significantly enhanced by stimulation with interferon (IFN)-γ in microglia. In turn, Gal-3 might activate the microglial M1 phenotype via IFN-γ and further elicit the production of proinflammatory cytokines by signalling cascades of the Janus kinase (JAK)/signal transducer and activator of transcription (STAT) pathway [68]. As an endogenous paracrine Toll-like receptor 4 (TLR4) ligand, endogenous Gal-3 binds to microglial TLR4, triggering a sustained M1 proinflammatory response under acute neuroinflammatory conditions [69]. Further study confirmed that Gal-3 increases the expression of proinflammatory markers, such as interleukin (IL)-1β, IL-6 and NOS2, in traumatic brain injury [70]. Gal-3 knockout diabetic mice showed decreased levels of Iba1 (+) microglia and inhibited neuroinflammation in the retina and optic nerve [71]. As one of the antibody-mediated effects in EAE mice, activation of microglia has opposing harmful and protective effects. On the one hand, Gal-3 is engaged in macrophage polarization mediated by IL-4; thus, its disease-exacerbating effect in EAE may be associated with the activation and inflammatory response of microglia [72, 73]. On the other hand, Gal-3 is induced in activated microglia that are implicated in clearance of cell debris and damaged axons and axon regeneration and remyelination, thus indicating the effective neuroprotection of Gal-3 in EAE mice [74].

Gal-3 is mainly produced by macrophages and can compete with TLR4 to bind lipopolysaccharide (LPS). Thus, Gal-3-LPS binding limits the interaction between Gal-3 and TLR4 and blocks downstream inflammatory cytokine production, suggesting that Gal-3 is a negative regulatory protein inhibiting LPS-induced inflammation. In contrast, Gal-3-deficient macrophages significantly activated the LPS-induced signalling pathway and downstream inflammatory cytokine production [75]. A study showed that the Gal-3/insulin-like growth factor 1 receptor (IGFR-1) interaction plays a vital role in IGF-mediated microglial proliferation in response to ischaemic injury. Furthermore, a lack of Gal-3 notably attenuates microglial activation and proliferation and induces IL-6 expression and JAK/STAT pathway activation [76].

LPS-activated microglia secrete sialidase (neuraminidase) to eliminate sialic acid from cell surface glycoproteins, which permits Gal-3 to bind the phagocytic receptor Mer tyrosine kinase (MerTK) and then promote opsonization and phagocytosis by microglia [77]. Gal-3 improves and lengthens the activity of KRas-GTP-dependent phosphatidylinositol 3-kinase, thereby inducing phagocytosis in microglia [78]. Similarly, Gal-3 activated phagocytosis of myelin to maintain myelin-debris clearance in autoimmune demyelinating diseases [79]. Interestingly, the expression of Gal-3 is only present in microglia that phagocytose degenerated myelin rather than in microglia that do not phagocytose degenerated myelin. Moreover, Gal-3 released by activated microglia can bind to bacteria and subsequently promote microglial phagocytosis of these bacteria [80]. After infection with a parasite, Gal-3 induced the M2 microglial phenotype to exhibit efferocytic clearance of neutrophils, confirming its neuroprotective roles in neuroinflammatory diseases [81]. As an advanced glycosylation end product (AGE)-binding protein, Gal-3 interacts with AGE to degrade its toxicity by fusing lysosomes [77].

### Gal-3 and AD

#### Gal-3 levels increased in AD

As the most common neurodegenerative disease, AD involves a complex pathological process that is distinguished by the presence of extracellular amyloid Aβ plaques and intraneuronal deposits of NFTs containing hyperphosphorylated tau protein. Nonsoluble and neurotoxic Aβ is derived from amyloid precursor protein (APP) through the consecutive action of β-secretase and γ-secretase enzymes and is deposited in brain tissue [82]. Increasing evidence suggests that microglia-mediated neuroinflammation plays an important role in AD pathogenesis [83, 84]. On the one hand, microglia help eliminate Aβ aggregation [85]. Aberrantly expressed cytokine levels and Aβ phagocytosis receptors result in an insufficient microglial phagocytic capacity and inefficient clearance of Aβ [86]. On the other hand, microglia promote Aβ accumulation by releasing neurotoxic proteases and inflammatory factors, such as IL-1, IL-4, IFN-γ, iNOS, and TNF-α. Multiple inflammatory factors overexpressed by activated microglia are related to Aβ cascade reactions during the occurrence and progression of AD [4, 87]. In recent years, many studies have confirmed that Gal-3 is a critical contributor to AD pathogenesis. Clinical and experimental studies have demonstrated the involvement of Gal-3 in AD.

Table 1 summarizes several studies that found relationships between Gal-3 and AD in humans. The longitudinal PROspective Study of Pravastatin in the Elderly at Risk (PROSPER) genotyped the rs4644, rs4652, and rs1009977 polymorphisms of the LGALS3 gene, encoding Gal-3, and found that the variant alleles were significantly associated with a decline in cognitive performance in 5804 elderly adults. Despite a lack of data on serum Gal-3, participants with a LGALS3 single nucleotide polymorphism (SNP) had significantly elevated C-reactive protein levels, which indicated that Gal-3 might induce cognitive dysfunction through inflammation [88]. Several prospective and cross-sectional studies found that serum [11] and cerebrospinal fluid (CSF) [89] Gal-3 levels were significantly higher in patients with AD than in age-matched healthy controls. Another clinical study further found that the expression of galactin-3 in the frontal lobe was increased in patients with AD, while Aβ oligomerization was enhanced [90]. Furthermore, serum Gal-3 levels significantly paralleled the severity of memory loss [90] and the stage of AD [91].

**Table 1:** Brief summary of clinical studies on the concentration and role of Gal-3 in AD

Study population	Locations	Method	Main findings	Authors
5804 participants aged 70–82 years were followed for 9–48 months	LGALS3 gene	MALDI-TOF MS	Genetic variation in the LGALS3 gene might be associated with cognitive function, and Gal-3 might influence cognitive function via the inflammatory response	Trompet et al. [88]
41 AD patients and 46 healthy subjects (controls)	Serum Gal-3 levels	ELISA	Patients with AD presented higher Gal-3 levels than healthy controls. Serum Gal-3 could be a potential a biomarker for AD diagnosis	Wang et al. [11]
31 AD patients and 50 healthy subjects (controls)	CSF and serum Gal-3 levels	ELISA	Serum and CSF galectin -3 levels in AD patients were higher than those in healthy controls. Serum Gal-3 concentration was positively correlated with the MMSE score	Ashraf et al. [89]
57 AD patients and 61 healthy subjects (controls)	Serum Gal-3 levels	ELISA	Serum galactin-3 levels might be positively correlated with the stage of AD and be a potential biomarker for the identification of AD	Yazar et al. [91]
4 AD patients and 4 healthy subjects (controls)	Frontal lobe tissue Gal-3 levels	Immunohistochemistry and western blot	Galactin-3 expression in the frontal lobe was increased in AD patients and paralleled Aβ oligomerization. Immunohistochemical results revealed colocalization of galactin-3 and amyloid plaques	Tao et al. [90]
6 AD patients and 5 healthy subjects (controls)	Cortex tissue Gal-3 levels	Immunohistochemistry and western blot	Gal-3 was increased in AD patient brains and colocalized with microglia associated with Aβ plaques	Boza-Serrano et al. [12]

Aβ, amyloid-β; AD, Alzheimer’s disease; CSF, cerebrospinal fluid; ELISA, enzyme-linked immunosorbent assay; Gal-3, galectin-3; MALDI-TOF MS, matrix-assisted laser desorption/ionization time-of-flight mass spectrometry

### Gal-3 induces neuroinflammation and neurodegeneration in AD

**Fig. 3:**
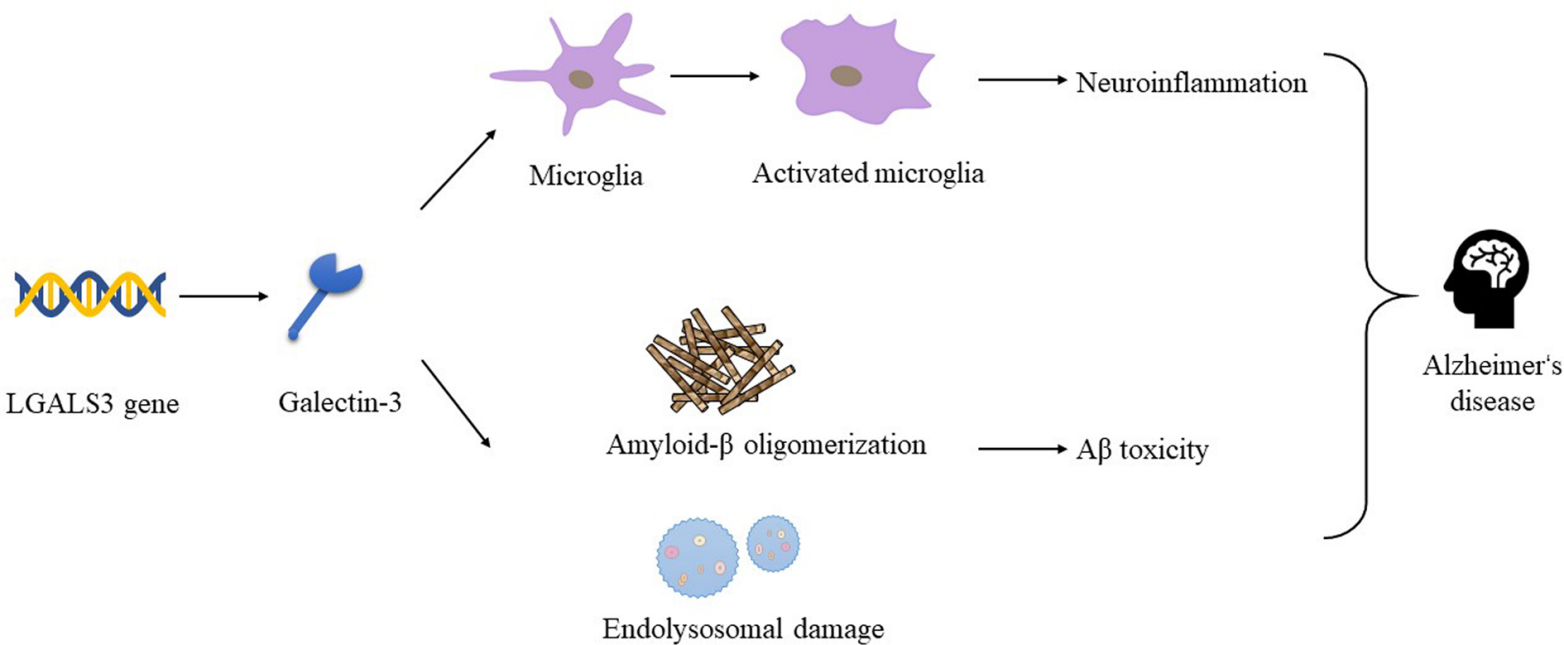
Pathogenic effect of galectin-3 (Gal-3) in Alzheimer’s disease

Previous studies have suggested that Gal-3 plays a dominant role in enhancing inflammation in the pathogenesis of AD (Fig. 3). In another study, Aβ_25-35_ was injected into the CA1 region of the rat hippocampus via a stereotactic method, which upregulated the expression of Gal-3 in activated microglia and astrocytes. Moreover, Gal-3 might modulate the neuroinflammatory response and neurodegeneration induced by administration of Aβ_25-35_ [92]. Consistently, Gal-3 in the brain was preferentially expressed in AD patients and a 5xFAD transgenic mouse model of familial AD and colocalized with Aβ plaque-associated microglia [12]. Gal-3 induced microglial activation accompanied by the production and release of proinflammatory factors, such as IL6, IL8 and TNFα. In Gal-3 knockout 5xFAD mice, the expression of proinflammatory microglia was decreased in vivo, amyloid plaques were reduced, and cognitive ability was improved. In addition, as an endogenous TREM2 ligand, Gal-3 directly interacts with TREM2 via its carbohydrate recognition domain to trigger microglia-associated immune responses through TLR- and TREM2/DAP12-dependent signalling [12].

Gal-3 overexpression strongly promoted Aβ oligomerization in C57Bl/6 WT mice injected intrahippocampally with Aβ, whereas Aβ oligomerization was reduced in Gal-3-knockout C57Bl/6 mice [90]. Moreover, there was an age-dependent increase in Gal-3 expression and endogenous Aβ oligomerization when evaluating the APP/PS1 transgenic mouse model of AD. Further analysis found that microglia-secreted Gal-3 interacted with Aβ directly, leading to Aβ oligomerization and Aβ toxicity [90]. Pathogenic Aβ and Tau are disseminated in neurons and microglia through endocytosis. Subsequently, the endolysosome is damaged, which releases Aβ and Tau into the cytoplasm and accelerates the process of seeded aggregates. Gal-3 also induces microglia to phagocytize Aβ monomers, which might be involved in the endocytosis and endolysosome damage mechanism of Aβ aggregates [93]. Table 2 summarizes the underlying molecular mechanisms of the Gal-3 pathogenic effect in AD.

**Table 2:** Experimental studies on the concentration and role of Gal-3 in AD

Animal models	Main findings	Authors
5xFAD mice and Gal-3 knockout mice	Gal-3 acted as an endogenous TREM2 ligand to modulate the proinflammatory response in AD	Boza-Serrano et al. [12]
Aβ_25-35_-induced Wistar rats	Neuroinflammation induced by the Aβ_25-35_ increased the expression of Gal-3 in astrocytes and microglia and damaged spatial memory	Ramírez et al. [92]
Gal-3 knockout mice and APP/PS1 mice	Overexpression of Gal-3 enhanced Aβ oligomerization through direct interactions with Aβ and inhibition of the degradation of Aβ	Tao et al. [90]

Aβ, amyloid-β; AD, Alzheimer’s disease; Gal-3, galectin-3; TREM2, triggering receptor expressed on myeloid cells 2

### Gal-3 as a biomarker

The plasma Gal-3 levels have been detected by ELISAs (enzyme-linked immunosorbent assays) in the majority of relevant clinical studies [11, 91]. This approach is simple and accessible for clinical practice. However, does this peripheral level accurately reflect the level in the tissue? This question is worthy of consideration. Gal-3 was overexpressed in the brain parenchyma [94] and CSF [89] in AD patients. Considering the blood–brain barrier breakdown in the AD pathological process [95], this phenomenon might lead to an elevation in Gal-3 levels in peripheral blood. One study on heart failure found no correlation between plasma Gal-3 and heart tissue Gal-3 levels [96]. Another study found that the CSF Gal-3 levels were not correlated with the CSF protein levels in neonatal hypoxic ischaemic injury, which provided reservations about the increased Gal-3 levels caused by blood flow across the blood–brain barrier [97]. There are several possible explanations for these differences. First, peripheral Gal-3 might reflect systemic inflammation. Second, the systemic and local functions of Gal-3 may be different. Third, temporal patterns of Gal-3 expression in the tissue and in the peripheral blood might be different. Thus, further investigations are needed to explain these conflicting phenomena.

### Gal-3 inhibitors and their therapeutic use for AD

Since Gal-3 recognizes and binds glycoprotein oligosaccharides through the carbohydrate recognition region, the pharmacological inhibitory effect of Gal-3 is mediated by CRD to inhibit the activity of the protein. Given the multiple effects of Gal-3 and its various localizations in tissues, such as intracellular, membrane-bound or extracellular, different inhibitors with different types of cellular uptake have been synthesized [98, 99]. At present, these inhibitors are generally classified into carbohydrate and peptidic noncarbohydrate types. Thiodigalactoside (TDG) monovalent derivatives, one of the most potent Gal-3 chemical antagonists, inhibited tumour growth by expressing the cancer-enhancing activities of Gal-3 [100]. Among Gal-3 inhibitors and modulators, GR-MD-02 (belapectin) has entered phase 2b studies for nonalcoholic steatohepatitis with cirrhosis and portal hypertension. This molecule has oligosaccharide chains containing galactose residues and binds to the central Gal-3, which plays a role in the development and progression of fibrosis [101]. Since the pathogenic role of Gal-3 in AD has been established, several hypotheses on its therapeutic use have been proposed [6].

For example, modified citrus pectin (MCP) is a natural polysaccharide extracted from citrus plants and has been extensively used in experimental studies as a classical Gal-3 inhibitor [102–104]. MCP prevented disruption of the blood–brain barrier and brain injury in a mouse model of subarachnoid haemorrhage, which indicates that Gal-3 plays a role in this brain injury model by regulating the inflammatory response [6]. In our recent study, MCP attenuated neuroinflammation, oxidative stress, and cognitive impairment in high-fat diet (HFD)/streptozotocin (STZ)-induced diabetic rats. In addition, MCP attenuated inflammation and oxidative stress in high glucose-stimulated BV-2 microglial cells [105]. Studies have shown that inhibiting Gal-3 can protect neurons from damage induced by inflammation, especially in the hippocampus and striatum [106]. Therefore, the inhibition of Gal-3 might be a new drug target for neurodegenerative diseases, such as AD.

Gal-3 might be a potential therapeutic target for mesenchymal stem cell-based treatment and interfere with the AD pathomechanism. In a recent study, human umbilical cord blood-derived mesenchymal stem cells (hUCB-MSCs) were transplanted into 5xFAD mice through an intracerebroventricular approach [107]. An interesting finding was that Gal-3 derived from hUCB-MSCs could reduce phosphorylated tau in in vitro and in vivo experimental models. Furthermore, the potential mechanism involved Gal-3 inhibition of hyperphosphorylation through GSK-3β mediation.

The protective or harmful effects of Gal-3 might depend on the different cell types expressing Gal-3 or the different responses to the stimulatory microenvironment [107, 108]. Hence, Gal-3 may have potential as a key biomarker in AD diagnosis and therapy and may improve neuronal function recovery.

### Gal-3 in other neurodegenerative diseases

#### Gal-3 in diabetes-associated cognitive impairment

Type 2 diabetes mellitus (T2DM) is a metabolic disease characterized by hyperglycaemia and glucose intolerance. Insulin resistance and insufficient insulin secretion due to impaired pancreatic β-cell dysfunction are two underlying mechanisms that lead to progressive clinical development. Recently, research has focused on cerebrovascular and neurodegenerative chronic complications of diabetes, such as diabetes-associated cognitive impairment [109]. In our previous study, we found that serum Gal-3 was significantly higher in T2DM patients with mild cognitive impairment (MCI) than in T2DM controls, even after adjustment for multiple potential confounders [110]. Similarly, Gal-3 levels in the serum and brain were increased in diabetic rats and were associated with neuroinflammation, oxidative stress, learning and memory impairment, while administration of MCP could reverse these outcomes [105]. Our findings indicated that Gal-3 might be a potential therapeutic target for cognitive impairment in diabetes.

### Gal-3 in amyotrophic lateral sclerosis

Amyotrophic lateral sclerosis (ALS), named motor neuron disease, is a neurodegenerative disease characterized by the degeneration of upper and lower motor neurons. The core pathological feature is intracellular inclusions in degenerating neurons and their axons. Several studies have noted that the expression of Gal-3 in the plasma [111] and cerebrospinal fluid [89] was increased in ALS patients and the ALS animal model SOD1(G93A), indicating that Gal-3 might be a candidate biomarker of ALS [112, 113]. Microglial expression of Gal-3 was present from the early presymptomatic stages and gradually increased to the end stage of the disease. Interestingly, Gal-3-positive microglia were confined in the ventral horns of the spinal cord at an advanced disease stage in the ALS animal model SOD1 (G93A) [114]. Gal-3 might play a protective role in ALS pathogenesis. Deficiency of Gal-3 contributed to microglial activation, inflammatory factor expression, and rapid disease progression in SOD1 (G93A) mice [115]. AGEs are responsible for neuroinflammation and neurodegeneration, and therefore, upregulation of Gal-3 expression by microglia may protect the tissue from AGE toxicity in amyotrophic lateral sclerosis (ALS) [115].

### Gal-3 in Parkinson’s disease

Parkinson’s disease (PD) is the second most common progressive neurodegenerative disorder worldwide. PD is characterized by progressive degeneration of the nigrostriatal dopaminergic pathway with substantial loss of substantia nigra pars compacta (SNpc) neurons and depletion of dopamine (DA) [116]. The pathologic characteristic of PD is the accumulation of α-synuclein, named Lewy bodies or Lewy neurites [117]. Clinical studies have shown that the serum Gal-3 level in idiopathic PD patients was higher than that in healthy controls, and it presented a significant correlation with Hoehn-Yahr scores, suggesting that Gal-3 is a biomarker for PD detection and the prediction of disease severity [118, 119]. A study noted that exogenous α-synuclein monomers or aggregates can activate microglia, induce upregulation of proinflammatory cytokines and participate in the neurodegenerative process of PD. When the expression of Gal-3 was inhibited by using siRNA or pharmacologically targeting Gal-3 activity, the α-synuclein-induced inflammatory response was alleviated. When α-synuclein was administered to wild-type mouse olfactory bulbs, activated microglia taking up α-synuclein developed a Gal-3-positive phenotype [94].

### Gal-3 in Huntington’s disease

Huntington's disease (HD) is an inherited disorder characterized by selective neuron loss in the striatum and cortex [120]. Gal-3 has been implicated in the inflammatory response leading to the pathogenesis of HD. An investigation showed that brain and plasma Gal-3 levels were higher in patients and mice with HD than in healthy controls [121]. Further investigation confirmed that Gal-3 was involved in the inflammatory response via NF-κB and the NOD-like receptor family pyrin domain-containing 3 (NLRP3) inflammasome-dependent pathways and that upregulated Gal-3 expression negatively modulated the clearance of damaged lysosomes in R6/2 microglia [121]. Thus, Gal-3 is a potent therapeutic target that regulates the microglia-mediated pathogenesis of HD.

## Conclusions

Gal-3 is a fascinating protein with a pleiotropic role in several physiological pathways. Numerous studies have shown that Gal-3 contributes to inflammation, microglial activation, remyelination, and neurodevelopment in neurodegenerative diseases. Major progress has been made in our comprehension of Gal-3 in the context of AD just a short time since the initial description of AD-associated Gal-3 abnormalities. This review summarizes Gal-3-induced microglia-mediated neuroinflammation in AD pathology. Clinical and experimental studies indicate that Gal-3 is crucial for the microglial response to the pathology of AD and that overexpression of Gal-3 may alter amyloid plaque aggregation and increase plaque-associated toxicity. The most prominent aspect of this molecule is that it can be successfully used as a risk marker of AD if measured in peripheral blood. The strong association of Gal-3 with the onset of AD and the prominent effects of Gal-3 on microglia in response to neuroinflammation suggest that Gal-3 inhibition for therapeutic purposes is an innovative and interesting strategy, although further clinical investigations are needed to clarify the potential for AD prevention.

## Data Availability

Not applicable.

## Abbreviations

Aβ: Amyloid-β
AD: Alzheimer’s disease
AGE: Advanced glycosylation end product
ALS: Amyotrophic lateral sclerosis
APP: Amyloid precursor protein
Bcl-2: B-cell lymphoma-2
bHLH: Basic helix–loop–helix
CLS: Collagen-like sequence
CNS: Central nervous system
CRD: Carbohydrate-recognition domains
CRE: cAMP-dependent response element
CSF: Cerebrospinal fluid
DA: Dopamine
ELISA: Enzyme-linked immunosorbent assay
ER: Endoplasmic reticulum
Gal-3: Galectin-3
GALIG: Gal-3 Internal Gene
GSK-3β: Glycogen synthase kinase-3β
HD: Huntington's disease
HFD: High-fat diet
hUCB-MSCs: Human umbilical cord blood-derived mesenchymal stem cells
IFN: Interferon
IGFR-1: Insulin-like growth factor 1 receptor
IL: Interleukin
LPS: Lipopolysaccharide
JAK: Janus kinase
KLF-3: Krüppel-like factor 3
MALDI-TOF MS: Matrix-assisted laser desorption/ionization time-of-flight mass spectrometry
MCI: Mild cognitive impairment
MCP: Modified citrus pectin
MerTK: Mer tyrosine kinase
MMPs: Matrix metalloproteinases
NFTs: Tau neurofibrillary tangles
NF-κB: Nuclear factor-κB
NLRP3: NOD-like receptor family, pyrin domain-containing 3
NTD: N-terminal domain
NTR: N-terminal region
PD: Parkinson’s Disease
PROSPER: PROspective Study of Pravastatin in the Elderly at Risk
Runx2: Runt-related transcription factor 2
SIE: Sis-inducible element
SNP: Single nucleotide polymorphism
SNpc: Substantia nigra pars compacta
STAT: Signal transducer and activator of transcription
STZ: Streptozotocin
T2DM: Type 2 diabetes mellitus
TDG: Thiodigalactoside
TLR4: Toll-like receptor 4
TREM2: Triggering receptor expressed on myeloid cells 2
TRPC1/4: Transient receptor potential canonical ¼
TTF-1: Thyroid-specific transcription factor 1
